# Supplementary material for: Content-rich biological network constructed by mining PubMed abstracts
Source: BMC Bioinformatics. 2004 Oct 8;5:147. doi: 10.1186/1471-2105-5-147 (PMC528731; doi:10.1186/1471-2105-5-147)
Supplement: Additional File 2 — The original results of the above study (non-essential files are deleted to keep the file size under the limit set by BMC bioinformatics). [file 1471-2105-5-147-S2.bz2 › chilibotAdditionalFile2/dip05/223ID7831290E739/html/SYK_LYN.html]

 


 **SYK** and **LYN** 
  
Found 264 abstracts in PubMed, retrieved 05.  
 

 What does Google say? 
 PDF only 
| .edu only 

---

**Interactive relationship** (e.g. stimulation, inhibition, etc)

**Stimulatory relationship**- Methyl dihydroxycinnamate DHC, a PTK inhibitor, was more selective for  **Syk**  than  **Lyn** , while staurosporine ST, a broad specificity protein kinase inhibitor, inhibited  **Lyn**  more than  **Syk** .  Ref: 12739161 Pflugers Arch, 2003
**Neutral relationship**

**Non-interactive relationship** (e.g. studied together, co-existance, homology, etc.)

- The activity of the kinases that phosphorylate the Fc receptor gamma chain, Fyn and  **Lyn** , as well as the tyrosine kinase  **Syk**  and phosphoinositide 3 kinase was also inhibited by quercetin in a concentration dependent manner, both in whole cells and in isolation.  Ref: 12871380 J Thromb Haemost, 2003
- In contrast, the tumor cells in all cases of lymphocyte predominance Hodgkin rad s disease were positive for Fyn,  **Syk** , BLNK and PLC gamma2, and  **Lyn**  immunostaining was seen in a minority of biopsies.  Ref: 12881301 BloodBlood, 2003
- We have reported previously that deoxygenation of sickle cells stimulates KCC and activates  **Syk**  a  **Syk**  family PTK, but not  **Lyn**  an Src family PTK.  Ref: 12739161 Pflugers Arch, 2003
- Stable expression of this  **Lyn**  PLCgamma2 was not accompanied by an increase in substrate hydrolysis in resting cells which followed stimulation and specifically required presence and or activation of  **Syk** , Btk, PI 3K but not BLNK, as established using deficient cell lines or specific inhibitors.  Ref: 12780340 Biochem J< MedlineTA>Biochem J, 2003
